# Glucagon-like peptide 1 decreases lipotoxicity in non-alcoholic steatohepatitis

**DOI:** 10.1016/j.jhep.2015.08.038

**Published:** 2016-02

**Authors:** Matthew J. Armstrong, Diana Hull, Kathy Guo, Darren Barton, Jonathan M. Hazlehurst, Laura L. Gathercole, Maryam Nasiri, Jinglei Yu, Stephen C. Gough, Philip N. Newsome, Jeremy W. Tomlinson

**Affiliations:** 1 NIHR Liver Biomedical Research Unit and Centre for Liver Research, University of Birmingham, Birmingham, UK; 2 CRUK Clinical Trials Unit, University of Birmingham, Birmingham, UK; 3 Centre for Endocrinology, Diabetes and Metabolism, Institute of Biomedical Research, School of Clinical and Experimental Medicine, University of Birmingham, Edgbaston, Birmingham, UK; 4 School of Sport, Exercise & Rehabilitation Sciences, University of Birmingham, Birmingham, UK; 5 Oxford Centre for Diabetes, Endocrinology and Metabolism, and NIHR Oxford Biomedical Research Centre, University of Oxford, Churchill Hospital, Oxford, UK

**Keywords:** Glucagon-like peptide 1, Non-alcoholic fatty liver, Insulin sensitivity, Adipose tissue, Lipolysis

## Abstract

**Background & Aims:**

Insulin resistance and lipotoxicity are pathognomonic in non-alcoholic steatohepatitis (NASH). Glucagon-like peptide-1 (GLP-1) analogues are licensed for type 2 diabetes, but no prospective experimental data exists in NASH. This study determined the effect of a long-acting GLP-1 analogue, liraglutide, on organ-specific insulin sensitivity, hepatic lipid handling and adipose dysfunction in biopsy-proven NASH.

**Methods:**

Fourteen patients were randomised to 1.8 mg liraglutide or placebo for 12-weeks of the mechanistic component of a double-blind, randomised, placebo-controlled trial (ClinicalTrials.gov-NCT01237119). Patients underwent paired hyperinsulinaemic euglycaemic clamps, stable isotope tracers, adipose microdialysis and serum adipocytokine/metabolic profiling. *In vitro* isotope experiments on lipid flux were performed on primary human hepatocytes.

**Results:**

Liraglutide reduced BMI (−1.9 *vs*. +0.04 kg/m^2^; *p <*0.001), HbA1c (−0.3 *vs*. +0.3%; *p* *<*0.01), cholesterol-LDL (−0.7 *vs*. +0.05 mmol/L; *p <*0.01), ALT (−54 *vs.* −4.0 IU/L; *p <*0.01) and serum leptin, adiponectin, and CCL-2 (all *p <*0.05). Liraglutide increased hepatic insulin sensitivity (−9.36 *vs*. −2.54% suppression of hepatic endogenous glucose production with low-dose insulin; *p <*0.05). Liraglutide increased adipose tissue insulin sensitivity enhancing the ability of insulin to suppress lipolysis both globally (−24.9 *vs*. +54.8 pmol/L insulin required to ½ maximally suppress serum non-esterified fatty acids; *p <*0.05), and specifically within subcutaneous adipose tissue (*p <*0.05). In addition, liraglutide decreased hepatic *de novo* lipogenesis *in vivo* (−1.26 *vs*. +1.30%; *p <*0.05); a finding endorsed by the effect of GLP-1 receptor agonist on primary human hepatocytes (24.6% decrease in lipogenesis *vs*. untreated controls; *p <*0.01).

**Conclusions:**

Liraglutide reduces metabolic dysfunction, insulin resistance and lipotoxicity in the key metabolic organs in the pathogenesis of NASH. Liraglutide may offer the potential for a disease-modifying intervention in NASH.

## Graphical abstract

**Figure:**
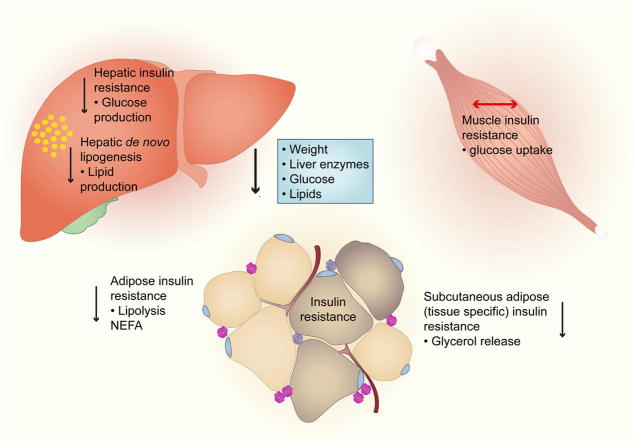

## Introduction

Non-alcoholic steatohepatitis (NASH) incurs a significantly increased risk of both liver- and cardiovascular disease (CVD)-related morbidity and mortality [1], yet, there remains a lack of safe and efficacious pharmacological treatments [2].

Insulin resistance (IR) in both liver and adipose tissue is believed to be a key driver in the pathogenesis of NASH [3]. Detailed metabolic studies, using ‘gold-standard’ euglycaemic clamps and/or stable isotope tracers, have demonstrated that patients with NASH have severe adipose IR, alongside increased hepatic IR [4], [5] and *de novo* lipogenesis (DNL) [6], [7]. Even though collectively these contribute to excess lipid accumulation in the liver, it is widely believed that the overspill of non-esterified fatty acids (NEFA) and release of triglyceride-derived toxic metabolites from adipose tissue lipolysis, form the primary lipotoxic insult in the pathogenesis of NASH and its extrahepatic complications including increased CVD-morbidity and mortality [1], [8]. In addition to driving intrinsic hepatic IR and inflammation, hepatic lipotoxicity is thought to further fuel the circulating pro-inflammatory milieu and IR status in NASH, which in turn contributes to the cycle of worsening adipose dysfunction and lipolysis [8]. Therefore, identifying pharmaceutical options that can target both liver and adipose IR as well as reduce hepatic exposure to the effects of lipotoxic metabolites may provide the best strategy to halt the progression of NASH and its clinical consequences.

Glucagon-like peptide-1 (GLP-1) analogues have been shown to improve glycaemic control, weight loss and in retrospective studies, liver enzymes in patients with type 2 diabetes [9], making them an attractive therapeutic option in NASH. Recent animal studies of NASH have supported these findings by demonstrating improvements in hepatic steatosis following GLP-1 therapy [10], [11], [12], [13], which in some cases was accompanied by reductions in oxidative stress [11], [14], [15] and fibrosis [16]. In particular, using euglycaemic clamp techniques, murine studies have shown that chronic GLP-1 administration improves insulin sensitivity and reduces hepatic glucose production [13], [17], [18]. Similar findings have been reported with short-durations of GLP-1 treatment ranging from single infusions up to 6-weeks in healthy volunteers [19] and in patients with type 2 diabetes [20]. Importantly, no such studies have been performed in the context of patients with NASH. The effects of GLP-1 on muscle insulin sensitivity in humans have been inconsistent albeit with most studies showing improvements in glucose disposal [19], [21], [22]. To date, there are no data that have examined the impact of GLP-1 treatment on human adipose insulin action *in vivo*.

The effect of GLP-1 analogues on metabolic dysfunction, most notably tissue-specific IR and hepatic DNL, in patients with biopsy-confirmed NASH is currently unknown. We therefore incorporated functional measures of lipid and carbohydrate flux at baseline and 12 weeks into the treatment regimen of our phase II, double-blinded, placebo-controlled randomised clinical trial, entitled ‘Liraglutide Efficacy and Action in NASH (LEAN) [23]’. Our aims were to determine the effect of 12-weeks treatment of liraglutide on tissue-specific IR (adipose, muscle, liver), hepatic DNL and adipose tissue function in patients with biopsy-defined NASH. In addition, we performed isotope tracer studies on primary cultures of human hepatocytes to establish if GLP-1 analogues have direct lipid-lowering effects, independent of changes in weight and glycaemic control as reported *in vivo*.

## Methods and materials

### Clinical study

The full clinical protocol of the LEAN trial (clinicaltrials.gov NCT01237119) has previously been described [23]. This was an investigator-initiated/led study, with charitable (Wellcome Trust, NIHR) and pharmaceutical (Novo Nordisk Ltd) support, with the University of Birmingham (UK) acting as the sole sponsor. The National Research Ethics Service (NRES) East Midlands – Northampton committee (UK) and the Medicines and Healthcare products Regulatory Agency (MHRA) approved all versions of the study protocol. All adult subjects gave informed written consent prior to participation.

#### Study participants

Consecutive adult patients from the Queen Elizabeth University Hospital Birmingham trial site only (UK), who met the eligibility criteria for the LEAN trial, were given the option of participation in the current experimental metabolic study. With the exception of the voluntary component, other aspects of study bias were minimised by incorporating the metabolic sub-study into the first 12-weeks of the randomised, double-blinded, placebo-controlled LEAN trial. The full eligibility criteria are listed in the published trial protocol [23]. All participants had a definitive diagnosis of NASH on liver biopsy within 6 months of the study, as defined by two independent liver histopathologists [2]. The participants were of adult age (18–70 years) and had a body mass index (BMI) ⩾25 kg/m^2^. Patients with co-existing type 2 diabetes were diet-controlled or were on a stable dose of metformin ± gliclazide for a minimum of 3 months prior to the study and had a HbA1c <9.0%. All patients with no previous diagnosis of type 2 diabetes underwent a 75 g oral glucose tolerance test.

#### Treatment groups

Patients who satisfied the eligibility criteria were randomly assigned on a 1:1 basis to once-daily (OD) subcutaneous injection of 1.8 mg liraglutide (Victoza®, Novo Nordisk A/S, Denmark) or liraglutide-placebo control (Novo Nordisk A/S, Denmark). To aid with gastrointestinal tolerability the dose was titrated by 0.6 mg every 7 days from a starting dose of 0.6 mg OD until the maximum dose of 1.8 mg OD was achieved.

#### Study design

At baseline and after 12-weeks of treatment all participants underwent paired 2-step hyperinsulinaemic euglycaemic clamps incorporating stable isotopes with concomitant subcutaneous adipose tissue microdialysis at the NIHR/Wellcome Trust Clinical Research Facility (WTCRF, Birmingham, UK) (Supplementary Fig. 1). A full description of such is detailed in the Supplementary Methods.

Participants were admitted to the WTCRF the evening (17.00 hours) before the euglycaemic clamp study. After a standardised meal, participants were fasted until completion of the clamp study, with the exception of drinking oral deuterated water (^2^H_2_O) to determine rates of DNL. At 08.00 hours the next morning fasting blood samples were taken and an adipose microdialysis catheter was inserted into the abdominal subcutaneous adipose tissue (SAT), prior to starting the 2-step hyperinsulinaemic euglycaemic clamp (as previously described [24]). Thereafter, microdialysate samples were collected into micro-vials (0.3 μl/min) every 30 minutes until the end of the clamp. After basal measurements, hepatic and peripheral (‘muscle’) insulin sensitivity were assessed with consecutive 2 hour infusions of insulin at 20 and 100 mU/m^2^/min, respectively. Fasting glycaemic concentrations were maintained (‘clamped’) with a concomitant variable infusion of 20% glucose enriched with U-[^13^C]-glucose (4%) throughout the hyperinsulinaemic phases. During the 6 hour clamp, steady state blood samples were taken at three time points in the final 30 minutes of the basal (90–120 min), low-dose (210–240 min) and high-dose insulin (330–360 min) phases.

### Data collection and analysis

Participant demographics and clinical/biochemical measures were recorded at baseline and after 12-weeks of treatment (see Supplementary Methods). Serum insulin (Mercodia, Sweden), NEFA (Zen-Bio, USA) and adipocytokines (Fluorokine® Multi-Analyte Profiling; R&D Systems, United Kingdom) were measured using commercially available kits, as previously described [24]. In addition, abdominal SAT microdialysate samples were analysed using a mobile photometric, enzyme-kinetic analyser (CMA Iscus Flex, Sweden) for interstitial glycerol concentrations.

#### Stable Isotope Mass Spectrometry analysis

The enrichment of U-[^13^C]-glucose in plasma (for hepatic endogenous glucose production (EGP) and glucose disposal (Gd) calculations) and deuterium (^2^H) in the body water pool/palmitate fraction of total plasma triglycerides (for DNL calculations) were determined by gas chromatography-mass spectrometry, as previously described [24], [25]*.*

#### Data definitions and calculations

Hepatic (hepatic EGP) and muscle (Gd) sensitivity were calculated by using modified versions of the Steele Equations. In order to quantify whole body lipolysis and adipose tissue insulin sensitivity, the Adipose-IR index and the insulin concentrations causing half-maximal suppression of serum NEFA (INS ½-max NEFA) were calculated for each participant using regression analysis [24], [25]. The INS ½-max NEFA was interpolated from the gradient in decline in circulating NEFA following the transition from basal, to low and then to high-dose insulin (maximal suppression) across the duration of the clamp. Similarly, the rate of glycerol release from SAT in response to fasting, low- and high-dose insulin was used to determine abdominal SAT lipolysis and insulin sensitivity. The percentage contribution of hepatic DNL to endogenous palmitate synthesis was determined by the incorporation of ^2^H_2_O in the palmitate present in the plasma total triglyceride pool, as previously described [24], [25].

### Human hepatocyte cultures

#### Cell culture

Cryopreserved primary human hepatocytes were purchased from Celsis IVT Technologies (Baltimore, Maryland, USA) and cultured at 37 °C in 5% CO_2_ using *Invitro*GRO™ CP medium with *Torpedo*™ antibiotic mix (Celcis, USA). All primary hepatocytes were from non-obese, non-diabetic male donors, with no history of liver disease or excess alcohol history (Supplementary Table 1). For all experiments, cells were serum-starved prior to treatment with either 10 nM/100 nM Exendin-4 (GLP-1 analogue; Sigma) or 5 nM insulin (positive control; Sigma). HuH7 cell lines were purchased from American Type Culture Collection (Manassas, VA, USA) and cultured at 37 °C in 5% CO_2_ using Dulbecco’s Modification of Eagle’s Medium (DMEM) (Invitrogen, UK) with 10% foetal calf serum (FCS) (Gibco®, UK), 1% non-essential amino acids, 2 mM glutamine, 10,000 units/l Penicillin and 10 mg/ml Streptomycin (Invitrogen, UK).

#### Hepatocyte DNL

The rate of DNL was measured by the amount of uptake of 1-[^14^C]-acetate into the lipid component of hepatocytes, as described previously [25]. After serum starvation, hepatocytes were incubated for 12 hours in serum free media containing Exendin-4 (10 nM or 100 nM). 1-[^14^C]-acetic acid [0.12 μCi/L; Perkin Elmer) with unlabeled sodium acetate [10 μM] was added to the treated serum-free media for an additional 6 hours. After incubation cells were washed and scraped into 250 ml of PBS (1×). The lipid fraction was recovered in Folch solvent (chloroform:methanol 2:1), the solvent was evaporated and the 14C radioactivity retained in the cellular lipid was determined by scintillation counting and expressed as disintegrations per minute (dpm)/per well.

#### Hepatocyte NEFA uptake and β-oxidation

The rate of NEFA uptake and β-oxidation was measured by the intracellular accumulation of 9,10-[^3^H]-palmitate and the conversion of 9,10-[^3^H]-palmitate (Perkin Elmer) to [^3^H] labeled-H_2_O, respectively; using a modification of the method described previously (Supplementary Methods) [26].

#### Oil Red O staining and triglyceride assays

HuH7 cells were exposed to DMEM containing 0.5% NEFA–free BSA and fat loaded with a mixture of 200 μM palmitic and 200 μM oleic acid (Sigma). After 12 hours, cells were washed with PBS (×1) and treated with 10 nM–100 nM exendin-4 for 12 hours. After further washing, cells were either fixed for Oil Red O staining (Sigma) or lysed for triglyceride quantification (Biovision #K622-100), using manufacturer’s instructions (Supplementary Methods).

### Statistical analysis

Continuous clinical and laboratory non-parametric variables were reported as median and interquartile range (IQR), unless stated. Area under the curve (AUC) analysis was performed using the trapezoidal method for interstitial glycerol release during the clamp. Baseline clinical and biochemical characteristics were compared between treatment groups using unpaired Wilcoxon signed-rank tests and Fisher exact tests for continuous and categorical variables, respectively. For each treatment group, comparison of baseline *vs.* post-treatment data was performed using paired Wilcoxon signed-rank tests. Unpaired Mann-Whitney tests were used to compare delta change (post-treatment minus baseline value for each subject) of variables in the placebo control *vs.* liraglutide. The significance level was set at *p <*0.05.

The *in vitro* data were expressed as mean ± SE. For comparison of two treatment arms, paired *t* tests were used. ANOVA with Dunnett’s post hoc analysis was used for comparisons of multiple doses and/or treatments. All analysis was performed using the GraphPad Prism 6.0 software package (GraphPad Software, Inc; California, US).

## Results

### Study participants

Fourteen patients were randomised to receive either placebo or liraglutide for 12-weeks. The two treatment groups were well matched with regards to baseline demographic characteristics, clinical, biochemical and clamp data (Table 1; Supplementary Tables 2 and 3). There were no significant differences between the placebo and liraglutide groups with respect to baseline NASH disease activity (median NAS [25th, 75th centile]: 4 [3,5] *vs*. 5 [4,5], *p *>0.05) and the presence of advanced fibrosis, defined as Kleiner F3-F4 (4 *vs*. 5 participants, *p *>0.99). There was no significant difference in the number of participants with type 2 diabetes (3 *vs*. 4), impaired (1 *vs*. 2) and normal glucose tolerance (3 *vs*. 1; *p = *0.478).

**Table 1:** **Changes in metabolic and liver parameters in participants receiving liraglutide and placebo for 12 weeks.**

Values are median (25th, 75th centiles). All blood parameters were in the fasting state. ∗*p* value, Wilcoxon pairs-signed-rank test. ∗∗*p* value, unpaired Mann-Whitney *U* test. There was no significant in baseline parameters between the two treatment groups.

### Clinical variables

Liraglutide decreased weight (−6.0 [−7.0,−5.0] kg; *p <*0.05), BMI (−1.9 [−2.8,−1.5] kg/m^2^; *p = *0.01), and total fat mass (−3.5 [−4.1,−1.8] kg; *p <*0.05) from baseline. In addition, waist circumference decreased (−8.0 [−10.0,−6.0] cm; *p <*0.05) as did abdominal fat mass (−1.6 [−2.3,−0.9] kg, *p <*0.05). In contrast, treatment with placebo resulted in no baseline changes with respect to anthropometric measures (Table 1). Glycemic control (HbA1c) improved with liraglutide treatment (−0.3 [−1.2,−0.1]%; *p <*0.05). Direct comparisons (median change from baseline) of the treatment effects of placebo and liraglutide are summarised in Table 1.

### Liraglutide improves liver biochemistry and markers of inflammation

Liraglutide improved liver enzymes from baseline, most notably aspartate transaminase (AST) (64 [40,87] *vs*. 37 [23, 39] IU/L; *p <*0.05) and alanine transaminase (ALT) (90 [36,137] *vs*. 36 [25, 74] IU/L; *p <*0.05). In contrast, no significant reductions were seen in the placebo group (Table 1). In direct comparison of delta changes from baseline, liraglutide significantly reduced AST, ALT and gamma-glutamyl transpeptidase (GGT) compared to placebo. Liraglutide reduced fasting serum leptin (12.7 [10.4,22.8] *vs*. 10.6 [8.39,13.5] ng/ml; *p <*0.05) and increased adiponectin (4.47 [3.68,6.47] *vs*. 6.28 [4.24,8.75] μg/ml; *p <*0.05) from baseline resulting in a significant reduction in the leptin-to-adiponectin ratio (3.15 [2.11,4.24] *vs*. 1.55 [1.18,2.85] ng/μg; *p <*0.05), implying improved adipose tissue function. Liraglutide reduced the levels of pro-inflammatory markers including CCL-2 (210 [203,238] *vs*. 203 [171,225] pg/ml; *p <*0.05) and high-sensitivity C-reactive protein (1.55 [0.63,3.89] *vs*. 0.46 [0.25,1.53] μg/ml; *p <*0.05). In direct comparison to the placebo group, circulating levels of adiponectin, leptin and CCL-2 were significantly lower in patients after liraglutide treatment. No significant reductions were seen in the placebo group (Table 2).

**Table 2:** **Effect of liraglutide and placebo on fasting serum adipocytokines and inflammatory markers.**

Adipocytokine profile performed on fasting serum at baseline and after 12-weeks treatment with either placebo or liraglutide. Values are median (25th, 75th centiles). ∗*p* value, wilcoxon pairs-signed-rank test. ∗∗*p* value, unpaired Mann-Whitney *U* test.

### Liraglutide improves systemic insulin sensitivity

Liraglutide reduced fasting serum glucose from baseline (5.48 [4.87,5.61] *vs.* 4.76 [4.65,4.83] mmol/L; *p <*0.05), whereas fasting serum glucose increased in patients receiving placebo (4.51 [4.43,7.17] *vs.* 4.78 [4.63,8.99] mmol/L; *p <*0.05). Liraglutide decreased fasting glucose from baseline compared to placebo (median baseline change: −0.65 [−0.91,−0.17] *vs.* 0.28 [0.01,1.34] mmol/L; *p <*0.001). Liraglutide did not change fasting insulin levels in comparison with baseline or with placebo (Table 1).

### Liraglutide improves hepatic insulin sensitivity

**Fig. 1:**
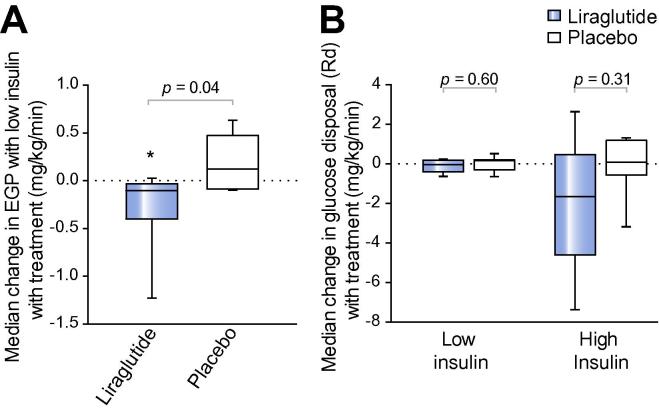
**Liraglutide significantly reduces hepatic insulin resistance, but has no effect on muscle insulin sensitivity.** (A) Tukey box-and-whisker plots highlight that liraglutide significantly increased the suppression of hepatic EGP with low-dose insulin compared to placebo. (B) Weight-adjusted Gd rates at low- and high-dose insulin phases of the euglycaemic clamp highlight that liraglutide did not change muscle insulin sensitivity (Gd) compared to placebo. ∗*p <*0.05 treatment *vs*. baseline (using paired Wilcoxon signed-rank tests). Unpaired Mann-Whitney tests were used to compare liraglutide *vs*. placebo.

Liraglutide increased the ability of low-dose insulin to suppress hepatic EGP (−43.2 [−47.4,−41.1] *vs*. −51.7 [−52.7,−49.5]%; *p <*0.05), consistent with improving hepatic insulin sensitivity, whereas there was no significant change in the placebo group (−49.2 [−51.0,−47.5] *vs*. −51.9 [−52.8,−37.0]%; *p = *0.94). The median difference in change in hepatic EGP with low-dose insulin was also greater with liraglutide than placebo (−0.10 [−0.4,−0.03] *vs*. 0.12 [−0.08, 0.47]%; *p <*0.05) (Fig. 1A).

### Liraglutide did not alter muscle insulin sensitivity

Liraglutide did not change weight-adjusted Gd rates in the low-dose (0.89 [0.61,0.97] *vs*. 0.76 [0.57,0.94] mg/kg/min; *p = *0.69) or high-dose insulin phases of the clamp (4.95 [2.49,6.50] *vs*. 3.28 [2.54,3.51] mg/kg/min; *p = *0.38). Similarly, patients treated with placebo had no differences in weight-adjusted Gd rates from baseline (Fig. 1B).

### Liraglutide improves whole body adipose insulin sensitivity and lipolysis

**Fig. 2:**
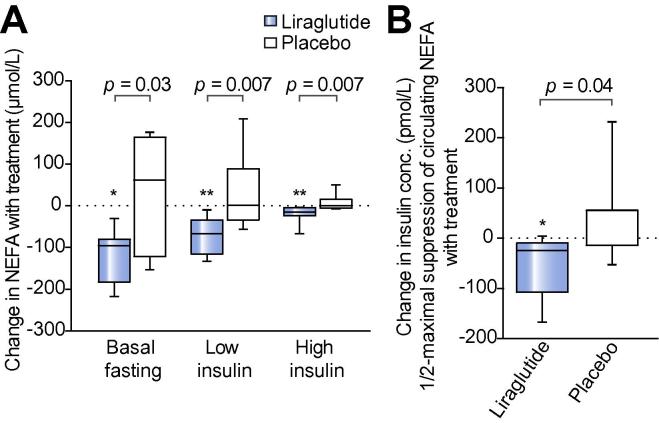
**Liraglutide significant reduces whole body lipolysis and adipose insulin resistance.** (A) Tukey box-and-whisker plots representing NEFA concentrations at the basal and hyperinsulinaemic phases of euglycaemic clamp. Liraglutide reduced NEFA at every phase of the clamp compared to placebo. (B) Tukey box-and-whisker plots representing the effect of liraglutide *vs.* placebo on insulin concentration required to achieve ½ maximal suppression of circulating NEFA (INS-½-max NEFA). ∗*p <*0.05 treatment *vs*. baseline (using paired Wilcoxon signed-rank tests). Unpaired Mann-Whitney tests were used to compare liraglutide *vs*. placebo.

Liraglutide significantly reduced circulating NEFA in the fasting (595 [425,656] *vs*. 452 [397,491] μmol/L; *p <*0.05) and hyperinsulinemic states, consistent with decreased whole body lipolysis. Similarly, there were no differences in the fasting (421 [397,628] *vs*. 534 [466,636] μmol/L; *p = *0.35) or hyperinsulinemic states in placebo treated patients. Circulating NEFA concentrations decreased with liraglutide *vs.* placebo treatment in the fasting (−95.8 [−183,−79.8] *vs*. +61.8 [−122,164] μmol/L; *p <*0.05), low-dose insulin (−66.8 [−115,−34.5] *vs*. +1.05 [−33.7,88.7] μmol/L; *p = *0.007) and high-dose insulin (−15.1 [−23.6,−4.1] *vs*. 0.0 [−5.52,16.1] μmol/L; *p = *0.007) phases of the euglycaemic clamps (Fig. 2A). In the fasting state, liraglutide significantly reduced the Adipose-IR index (8.42 [5.02,9.88] *vs.* 6.26 [4.41,7.28] mmol/L.uU/L; *p <*0.05), consistent with decreased adipose IR; there was no change in the placebo arm (8.03 [5.34,15.8] *vs*. 10.2 [7.67,12.5] mmol/L.uU/L; *p = *0.38). Similarly, liraglutide significantly reduced INS-½-max NEFA, with no reported change with placebo. Furthermore, liraglutide significantly reduced INS-½-max NEFA as compared to placebo (−24.9 [−107,−9.73] *vs*. +54.8 [−14.4,56.0) pmol/L; *p <*0.05), consistent with enhanced anti-lipolytic action of insulin following liraglutide treatment (Fig. 2B).

### Liraglutide improves subcutaneous adipose insulin sensitivity and lipolysis

**Fig. 3:**
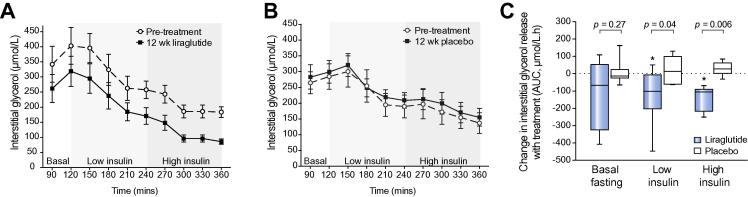
**Liraglutide reduces abdominal SAT lipolysis and insulin resistance (IR).** Line graphs (A, B) represent the mean ± SE concentrations of glycerol in the interstitial fluid measured from the SAT of each participant using *in situ* microdialysis throughout the 6 hour euglycaemic clamp. Liraglutide (A) decreased glycerol release throughout the clamp, whereas there were no clear differences after placebo treatment (B). (C) Tukey box-and-whisker plots (area under the curve analysis) highlight that liraglutide significantly reduced glycerol release from SAT in response to both low-dose and high-dose insulin compared to placebo, representing decreased abdominal SAT IR. ∗*p <*0.05 treatment *vs*. baseline (using paired Wilcoxon signed-rank tests). Unpaired Mann-Whitney tests were used to compare liraglutide *vs*. placebo.

Liraglutide reduced the rate of glycerol release from SAT in response to both low-dose (454 [347,504] *vs*. 321 [245,418] AUC.μmol/L.hr; *p <*0.05) and high-dose insulin (307 [214,360] *vs*. 194 [106,221] AUC.μmol/L.hr; *p <*0.05), consistent with increased abdominal SAT insulin sensitivity (Fig. 3A). Treatment with placebo had no effect on the rate of glycerol release into the interstitial fluid at either low-dose (352 155,437] *vs*. 348 [287,420] AUC.μmol/L.hr; *p = *0.68) or high-dose insulin (226 [71.3,372] *vs*. 237 [133,409] AUC.μmol/L.hr; *p = *0.11) (Fig. 3B). Furthermore, liraglutide significantly reduced the rate of glycerol release from SAT when compared to placebo (Fig. 3C). Despite normalizing the rate of glycerol release from SAT to changes in truncal fat mass (kg), the effect of liraglutide on abdominal SAT insulin sensitivity remained; thereby implying that the effect was independent of a tissue mass effect (Supplementary Fig. 2).

### Liraglutide reduces hepatic DNL *in vivo*

**Fig. 4:**
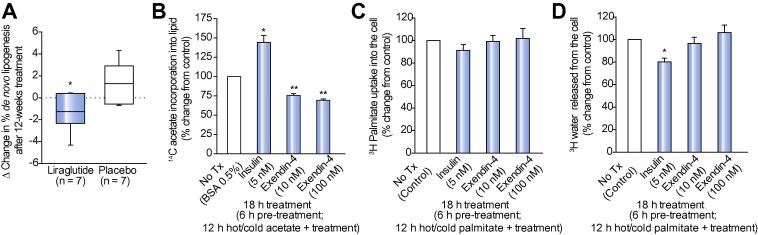
**GLP-1R analogues significantly reduce hepatic DNL *in vivo* and *in vitro*, respectively.** (A) Tukey box-and-whisker plots demonstrate that 12 weeks treatment with liraglutide significantly reduces hepatic DNL compared to placebo in patients with NASH (data ∗*p <*0.05 *vs*. placebo). (B) Exendin-4 significantly reduces DNL in primary human hepatocytes in culture. DNL was defined by the amount of ^14^C acetate incorporated into intracellular lipid in primary human hepatocytes. Exendin-4 has no effect on NEFA uptake (^3^H-palmitate taken up by the cells) (C) and β-oxidation (amount of 3H water released from the cells) (D) in hepatoma cell lines (HuH7). *In vitro* data in (B, C, D) are presented as mean ± SE percentages of the untreated controls. Untreated control was DMEM with 0.5% BSA. Insulin 5 nM served as a positive control. *In vitro* experiments were performed four times with each treatment in quadruplicate. ∗*p <*0.05 *vs*. untreated control.

The percentage contribution of hepatic DNL to total endogenous palmitate synthesis did not significantly change from baseline with either placebo (5.24 [4.42,6.90] *vs*. 6.85 [4.43,9.58]%; *p = *0.22) or liraglutide (4.87 [4.38,5.66] *vs*. 3.26 [2.58,4.85]%; *p = *0.15). However, liraglutide reduced hepatic DNL when compared directly to placebo (median change: −1.26 [−2.34,−0.40] *vs*. +1.30 [−0.56,2.91]%; *p <*0.05) (Fig. 4A).

### GLP-1 receptor (GLP-1R) analogues reduce hepatocyte steatosis *in vitro* through a reduction in hepatic DNL

Supporting the *in vivo* observations, GLP-1 receptor analogues (10 nM exendin-4) decreased ^14^C-acetate incorporation into intracellular lipid in both HuH7 cells (49.4 ± 7.1% decrease *vs*. control; *p <*0.05, n = 4) and primary human hepatocytes (24.6 ± 2.8% decrease *vs*. control; *p <*0.01, n = 4) in a concentration-dependent manner (100 nM exendin-4: HuH7, 70.5 ± 2.4%; primary hepatocytes, 30.7 ± 2.0% decrease *vs.* control; *p <*0.01 for both cell types) (Fig. 4B).

Exendin-4 (10 nM or 100 nM) had no effect on ^3^H-palmitate uptake (NEFA uptake) when compared to untreated cells (10 nM: 99.1 ± 5.7; 100 nM: 101.9 ± 8.6% of controls; *p *>0.05 for both doses, n = 4) (Fig. 4C). As expected, insulin (5 nM) decreased β-oxidation, however exendin-4 treatment (10 nM or 100 nM) did not change rates of β-oxidation (10 nM: 96.5 ± 5.9; 100 nM: 106.2 ± 7.0% of controls; *p *>0.05 for both concentrations, n = 4) (Fig. 4D).

**Fig. 5:**
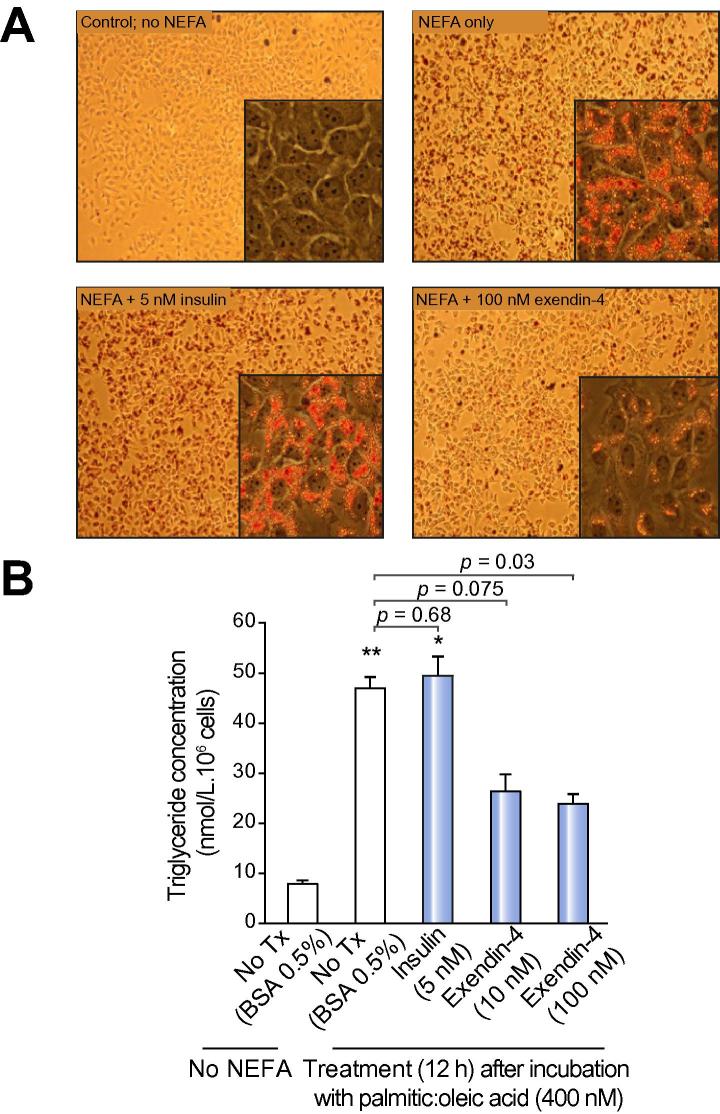
**GLP-1R analogue, exendin-4, has direct anti-steatotic effects on hepatocytes *in vitro*.** Exendin-4 (100 nM) reduces triglyceride content of NEFA-loaded HuH7 cells, as represented by (A) Oil Red O Staining (original magnification 10×, 40×) and (B) colorimetric triglyceride quantification assay. ∗*p <*0.05, ∗∗*p <*0.01 *vs*. untreated cells. (This figure appears in colour on the web.)

100 nM exendin-4 treatment (12 h) decreased intracellular triglyceride content, as demonstrated by a reduction in Oil Red O staining (Fig. 5A) and formal triglyceride quantification (10 nM exendin-4: 46.9 ± 2.4 *vs*. 26.5 ± 3.5 nmol/L/10^6^ cells; *p = *0.075; 100 nM exendin-4: 46.9 ± 2.4 *vs*. 23.9 ± 2.2 nmol/L/10^6^ cells; *p <*0.05) (Fig. 5B).

## Discussion

Therapies that protect the liver from the effects of lipotoxicity are believed to be essential for preventing the progression of NASH to end-stage disease [8]. Our experimental medicine study dissects the mechanism of action of the GLP-1 analogue, liraglutide, in patients with NASH. Liraglutide improved both hepatic and global/localised adipose insulin sensitivity, thereby reducing the amount of lipotoxic metabolites and pro-inflammatory mediators in the circulation. In addition, liraglutide reduced hepatic DNL *in vivo*, a key component of hepatic lipid accumulation in NASH. Supporting this clinical observation, our *in vitro* isotope studies indicated that GLP-1R analogues also have an anti-lipogenic action on hepatocytes, which cannot be attributed to weight loss.

Liraglutide decreased fasting glucose, although changes in Gd (during hyperinsulinaemia) did not change significantly, and this is in agreement with the published literature that has shown inconsistent actions of GLP-1 on skeletal muscle insulin action [19], [21], [22], [27]. These data would suggest that sites other than skeletal muscle (namely liver and adipose tissue) are the key targets for the beneficial effects of liraglutide in this study.

Our study has shown significant metabolic improvements in the liver following treatment with liraglutide. We have previously shown that the suppression of hepatic EGP by insulin is blunted in patients with NASH [24] and data from our current study indicate that liraglutide is able to restore hepatic insulin sensitivity in patients with NASH. It is likely that this is the major contributor to the improvement in fasting glucose levels. These effects on hepatic insulin sensitivity are supported by GLP-1 studies in patients with [20], [28] and without [19], [21] type 2 diabetes, albeit that the duration of treatment in these studies was shorter and the underlying status of liver disease was unknown. The mechanisms by which GLP-1 induce hepatic insulin sensitivity are not clear, with earlier studies attributing it to GLP-1’s inhibitory effect on glucagon and subsequent decrease in hepatic glycogenolysis and gluconeogenesis [29]. In contrast, recent studies in rodents and humans have shown that the effect is likely independent of endogenous pancreatic hormones [21], [27].

Hepatic DNL is a major contributor to lipid accumulation in the context of NAFLD [6]. We have provided the first *in vivo* evidence that GLP-1 based therapy can decrease DNL in patients with biopsy-proven NASH. Although the basal percentage contribution of hepatic DNL to total hepatic lipid content is lower than previously reported (4.9%) [6], [7], we still observed a significant reduction in the percentage of hepatic DNL with liraglutide *vs.* placebo. Supporting our clinical observations in patients with NASH, we found that GLP-1R agonist (exendin-4) significantly reduced hepatic DNL (as measured by the incorporation of ^14^C-acetate into cellular lipid) in both hepatoma cell lines and primary cultures of human hepatocytes. Our data are supported by weight neutral studies in rodents [12], [30] and the reduced gene expression of key enzymes involved in hepatocyte DNL including fatty acid synthetase, acetyl-CoA carboxylase 1, sterol regulatory element-binding protein 1 after GLP-1 treatment in culture [10], [14], [31]. Collectively, these data highlight the possibility that the effects of GLP-1R agonists on DNL can occur in the absence of weight loss. However, whether these effects are initiated directly via the transmembrane GLP-1R remains uncertain; largely as a result of discrepant reports of receptor expression on human hepatocytes [32].

Gene expression studies have highlighted the potential of GLP-1 to drive lipid oxidation *in vitro* [10], [14], [31], however, we did not observe a functional effect of GLP-1R agonism on β-oxidation (or fatty acid uptake) in hepatocytes using radio-labeled tracers. This implies that the majority of the anti-steatotic effect of GLP-1R agonism on hepatocytes is via DNL, rather than β-oxidation or NEFA uptake. In our cell cultures systems, GLP-1R agonism was able to decrease total lipid accumulation in the absence of insulin and it is possible that this may protect the hepatocyte from oxidative (endoplasmic reticulum) stress and subsequent cellular death *in vitro* [11] in a similar manner to observations made in pancreatic β-cells.

The beneficial effects of GLP-1 therapy are not confined to the liver and in our study we observed significant improvements in markers of adipose tissue function. The delivery of adipose tissue derived NEFA is the major factor driving lipid accumulation in NASH [6]. Furthermore, there is a growing body of evidence to suggest that adipose IR and subsequent excess release of NEFA into the circulation are linked with the onset of peripheral and hepatic IR [33], and progressive liver injury [4]. Therefore, treatments that are able to target adipose tissue dysfunction have the potential to impact upon the progression of NASH and its associated cardiovascular risk.

In the current study, liraglutide decreased circulating NEFA levels in the fasting state and enhanced insulin-mediated suppression of lipolysis. Rodent studies have reported similar effects on fasting NEFA levels [15], [17], but have not examined the impact upon insulin action to suppress lipolysis. Zander *et al.* have shown that 6 weeks of continuous subcutaneous GLP-1 infusion in patients with type 2 diabetes resulted in significant reductions in fasting and post-prandial NEFA levels [20]. In contrast, Seghieri and colleagues found no effect of GLP-1 on lipolysis, as determined by free fatty acid concentrations and tracer isotope studies [27]. This discrepancy may be explained by: duration of the treatment (i.e. 8 hours *vs.* 12-weeks therapy in our study); administration of treatment (i.e. intravenous native GLP-1 *vs.* subcutaneous GLP-1 analogue in our study); and the fact that the Segieri *et al.* utilised healthy volunteers, as opposed to patients with biopsy-confirmed NASH; and the reliance on global lipolysis readouts rather than localised abdominal adipose (SAT) tissue sampling (i.e. microdialysis). In rodent pre-adipocytes, GLP-1 augments insulin signaling through increased phosphorylation of IRS-1 and Akt, which in turn leads to enhanced glucose uptake [34], [35]. A direct action of GLP-1 to regulate lipolysis in the absence of insulin has not been a consistent *in vitro* finding and may reflect variations in dosage and culture conditions [35], [36], [37]. Similarly to hepatocytes, the presence of the GLP-1R in human adipocytes remains controversial. Interestingly, Vendell and colleagues reported that GLP-1R expression (mRNA, protein) is greatest in adipocytes isolated from patients with morbid obesity and in particular, IR [35]. The adipose tissue interstitial fluid analysis that we have performed would further endorse the concept of adipose tissue insulin sensitization by GLP-1 in a tissue where dysfunction is believed to be an important contributor to the pathogenesis and progression of NASH [4], [5], [24], [38]. Whilst we did not measure adipose tissue blood flow, previous studies have shown that it is not altered by GLP-1 and this would therefore not compromise out interpretation of our data, which mirrors changes in circulating NEFA levels [39]. Through sensitization of abdominal SAT to insulin, it is likely that liraglutide can increase its buffering capacity in response to external insults, for example as seen in calorific excess. As a result, the liver and other metabolically active organs may be protected from the overspill of lipotoxic metabolites into the systemic circulation.

Changes in the circulating pro-inflammatory milieu associated with NASH also suggest improved adipose tissue function following GLP-1 analogue therapy. Although adipose tissue biopsies were not performed as part of our study protocol, reductions in adipose tissue inflammation have been observed in rodents treated with DPP-IV inhibitor that increase endogenous GLP-1 levels [12]. Notably, we demonstrated increased levels of circulating adiponectin following liraglutide treatment, which were accompanied by corresponding reductions in circulating leptin levels. Furthermore, the leptin-to-adiponectin ratio was reduced to levels similar to those found in healthy volunteers [24]. Importantly, liraglutide has recently been shown to increase the expression and secretion of adiponectin from adipose tissue in a rodent model of NASH [18]. Furthermore, increases in circulating adiponectin, either via direct supplementation or secondary to other therapies have been implicated in the resolution of lipotoxic liver injury and fibrosis in humans [40]. Although our data do not allow us to be categorical, it is highly likely that the beneficial effects of liraglutide on adipose tissue metabolism (alongside the direct actions on the liver) will contribute to improved hepatic metabolic function and potential improvement in NASH.

The current study has recognised limitations. Firstly, even though clamp techniques are the recognised gold-standard for assessment of tissue-specific insulin sensitivity, the small sample size highlights the practical challenges of incorporating optional paired studies in a randomised-controlled clinical trial. Although the sample size limits statistical sub-group analysis (i.e. stage of fibrosis, diabetes status), the fact that 5/7 (71%) patients in the liraglutide arm had advanced fibrosis (F3/F4) highlights that improvements seen in adipose dysfunction, lipogenesis and insulin sensitivity are not limited to earlier stages of fibrosis. Indeed, larger powered studies are now warranted to validate our findings and investigate which phenotypic variables (i.e. fibrosis stage, presence of diabetes) influence the metabolic response to liraglutide. Secondly, is the confounding issue of weight loss in our study; patients lost approximately 6% of their initial starting body weight in the liraglutide treated group. This has made mechanistic interpretation of all data from studies that have used GLP-1 based therapy problematic. It is plausible that our observations could be explained by significant weight loss, however, rodent (weight neutral) studies, together with our *in vitro* data suggest that there are actions of GLP-1, which are not entirely dependent on weight loss. Throughout our *in vitro* studies, exendin-4 (exenatide) was selected over liraglutide as the therapeutic of choice, due to its commercial availability (in 2009) and stability in cell culture. Therefore future studies would usefully validate our findings with liraglutide in cell culture.

When this study was designed liraglutide was only available at the 1.8 mg dose, and since then the higher dose of 3.0 mg has been approved for weight management in the presence or absence of diabetes. It is plausible that the higher dose of liraglutide could provide greater efficacy in the setting of NASH and multi-organ IR, however this requires confirmation in future studies and should be weighed against the possibility of greater side-effects.

In summary, the data from this experimental study demonstrate the possible mechanisms by which liraglutide mediates its beneficial clinical and metabolic effects in patients with NASH. Specifically, liraglutide enhanced hepatic insulin sensitivity and decreased lipogenesis, which in part was not dependent on weight loss. In parallel, liraglutide increased adipose insulin sensitivity, thereby reducing the lipotoxic capabilities of dysfunctional fat tissue in NASH. This class of agent therefore has the potential to offer a novel therapeutic approach to the treatment of NASH and its CVD risk profile.

## Conflict of interest

PNN and MJA have received an educational grant and free trial drug supply from Novo Nordisk for conduct of the LEAN trial of liraglutide in NASH. PNN has received honoraria for lectures given on behalf of Novo Nordisk. SCG has served on advisory boards for Novo Nordisk, Eli Lilly, Sanofi Aventis and Takeda, and has received honoraria for lectures given on behalf of Novo Nordisk, Eli Lilly, Sanofi Aventis, Takeda and GSK. DH, KG, JMH, JY, MN, LLG and JWT have no conflict of interests to declare.

## Authors’ contributors

MJA, SCG, PNN and JWT had the original concept and contributed to the design of the study protocol. MJA and DB (senior trials coordinator) submitted all the National ethics, MHRA, local R&D and trial registration applications. MJA, with the assistance of DH, KG, JMH and JWT, performed the human clamp experiments and generated all of the clinical data for the manuscript. MJA, JY, and JWT analysed the human isotope data. MJA, with the assistance of MN and LLG performed the *in vitro* experiments. MJA and JWT performed the statistical analysis blinded to the randomised treatment. MJA and JWT wrote the first draft of the manuscript, and all authors reviewed the final version. MJA, PNN and JWT are guarantors.
