# Supplementary material for: Glucagon-like peptide 1 decreases lipotoxicity in non-alcoholic steatohepatitis
Source: J Hepatol. 2016 Feb;64(2):399–408. doi: 10.1016/j.jhep.2015.08.038 (PMC4713865; doi:10.1016/j.jhep.2015.08.038)
Supplement: Supplementary data [file mmc1.pdf]

Glucagon-like peptide 1 decreases lipotoxicity in non-alcoholic steatophepatitis

Matthew J Armstrong, Diana Hull, Kathy Guo, Darren Barton, Jonathan M Hazlehurst, Laura L Gathercole, Maryam Nasiri, Jinglei Yu, Stephen C Gough, Philip N. Newsome, Jeremy W Tomlinson

Table of content

Supplementary material and methods……………………………………………2

Supplementary Table 1………………………………………………………………7

Supplementary Table 2………………………………………………………………8

Supplementary Table 3………………………………………………………………10

Supplementary Fig. 1……………………………………………………………...12

Supplementary Fig. 2……………………………………………………………...13

Supplementary material and methods:

Study design (in detail):

Hepatic de novo lipogenesis (DNL): At 17.00 hours, total body water was estimated by bioimpedance (Tanita BC418MA, Amsterdam, NL). A standardised meal (carbohydrate 45g, protein 23g, fat 20g) was provided at 17.00 hours, after which participants remained fasted until the end of the clamp the next day. To determine rates of *de novo* lipogenesis (DNL), participants were given oral deuterated water, $^{2}H_{2}O$ (3g/kg total body water in 2 divided doses), at 18.00 hours and 22.00 hours followed by ad libitum drinking water enriched with 0.4% $^{2}H_{2}0$.

2-step hyperinsulinaemic euglycaemic clamp: At 08.00 hours the next morning, fasting blood samples were taken prior to starting the 2-step hyperinsulinaemic euglycaemic clamp. Arterialised blood was sampled to determine the blood glucose concentration at which to maintain the participant throughout the study using an YSI 2700 machine (YSI life sciences, UK). An intravenous bolus of U-[^{13}C]-glucose (2mg/kg body weight; CK gas limited, Hook, UK) was administered over 1 minute followed by a constant infusion rate (0.02mg/kg/min) for 6 hours until the end of the clamp. Steady state blood samples were taken at 3 time points during the final 30 minutes of the 2-hour basal phase. At 10.00 hours, low-dose insulin (Actrapid; Novo Nordisk, Copenhagen, Denmark) was infused at 20mU/m^{2}/min. At 10.04 hours a

concomitant infusion of 20% glucose enriched with U-[^{13}C]-glucose to 4% was commenced. Arterialised blood samples were taken at 5-minutely intervals and the 20% glucose infusion rate changed to maintain (‘clamp’) fasting glycaemic levels. Steady state blood samples were taken at 3 time points in the final 30 minutes of the 2-hour low-dose insulin infusion. The insulin infusion rate was then increased to 100mU/m^2/min (high-dose) for 2 hours with sampling as described above.

Adipose microdialysis: A microdialysis catheter (CMA microdialysis, Solna, Sweden) was inserted after local anaesthetic (5ml 1% lignocaine) into the abdominal SAT (minimum depth 1cm), 10cm lateral to the umbilicus, prior to commencing the clamp. Thereafter, micro-dialysate samples were collected into micro-vials (0.3µL/min) every 30 minutes until the end of the clamp.

Data collection and analysis: Systolic and diastolic blood pressure (*average of 2 readings*), waist circumference, weight, height, BMI and bioimpedance (Total body/truncal fat mass) were measured. Fasting blood samples (0800 hours) were analysed for full blood count (FBC) urea and electrolytes (U&E), liver function tests (LFTs), thyroid stimulating hormone (TSH), lipid profile, glycolated haemoglobin (HbA1c) and plasma glucose using standard laboratory methods (Roche Modular system, Roche Ltd, Lewes, UK). The adipocytokine profiling included adiponectin, leptin, resistin, tumour necrosis factor-alpha (TNF-α), high sensitivity C-reactive protein (hs-CRP), interleukin-6 (IL-6), chemokine ligand 2(CCL-2), CCL-4 and CCL-5.

Hepatocyte NEFA uptake and β-oxidation: The rate of NEFA uptake and β-oxidation was measured by the intracellular accumulation of 9,10-[^{3}H]-palmitate and the conversion of 9,10-[^{3}H]-palmitate (Perkin Elmer) to [^{3}H] labelled-H_2O, respectively; using a modification of the method described previously [27]. After serum starvation, cells were incubated for 6 hours in low-glucose (1g) serum-free media (DMEM) containing Exendin-4 (10 nM or 100 nM). The cells were then incubated at 37 ^{0}C for a further 12 hours with low-glucose serum free media containing exendin-4 and 0.12 μCi/L 9,10-[^{3}H]-palmitic acid (Perkin Elmer) with unlabelled 'cold' palmitate (total concentration = 100 μM). After incubation, cell lysate was recovered and intracellular ^{3}H radioactivity (i.e. amount of palmitate uptake) was determined. The incubation medium was also recovered and precipitated with an equal volume of 10% tricholoroacetic acid. The aqueous component of the supernatants was extracted with 2:1 chloroform:methanol solution. The ^{3}H radioactivity released into the media (i.e. rate of β-oxidation) was determined by scintillation counting and expressed as dpm/per well.

Oil red O Staining: Cells were fixed with formalin for 5 mins, washed with PBS (x1) and then incubated with 450 μL 60% isopropanol (Sigma) for 5 mins. 450 μL Oil Red O working solution was then added to each well and incubated for 45 minutes with gentle rocker. Of note, Oil Red O working solution was made by dissolving and incubating 0.25g of Oil Red O powder (Sigma) in 50 ml 100% isopropanol overnight at 37°C, followed by filtering the

excess solute off. After removal of the oil red solution, a further 450 μL of 60% isopropanol was added and left for 5 mins. After which the cells were washed three times with 450 μL of deionised water and visualised using a 40X microscope. Experiments were carried out three times in triplicate.

Triglyceride quantification assay: The assay was performed in keeping with the manufacturers instructions (Biovision #K622-100). In brief, Huh 7 cells were lysed in 1ml 5% NP-40 lysis buffer, placed in an eppendorph and heated to 80°C in a water bath until the solution went cloudy. The cell suspension was then cooled to room temperature and then re-heated to ensure the triglyceride had solubilised. The sample was then centrifuged at 14000 rpm (2 min) to remove insoluble material and then diluted 5-fold with sterile deionised water. Of note, 10-fold dilution was attempted but resulted in undetectable levels of triglyceride in the non-NEFA control. A Standard curve was prepared by diluting 2mM triglyceride standard with the assay buffer provided to generate 50 μL of standards at concentrations of 0, 2, 4, 6, 8 and 10nM/well. 2μL of lipase was then incubated at room temperature for 20 min with 50 μL of either sample or standard in a 96-well plate to convert triglyceride to glycerol and NEFA. 50 μL of triglyceride reaction mix (46 μL assay buffer, 2 μL triglyceride probe, 2 μL triglyceride enzyme mix) was added to each well and incubated for 60 mins in the dark. The optical density of each well was determined using a microplate reader set to 570 nm. A linear response of absorbance versus triglyceride standards was observed

(R^2=0.950). Experiments were carried out three times in triplicate and results were expressed in nmol/L per 10^6 cells.

Supplementary Tables:

Supplementary Table 1: Donor characteristics and cell viability of primary hepatocytes.

Donor characteristics	Donor (LOT No.)			
	**QOQ**	**LTG**	**CPQ**	**FOP**
Age (years)	66	56	31	62
Gender	Male	Male	Male	Male
Ethnicity	Caucasian	Caucasian	Caucasian	Caucasian
BMI (Kg/m^2)	29.1	27.8	25.9	29.0
Viral Status (HBV/HCV/HIV/CMV)	Negative	Negative	Negative	Negative
Alcohol history (drinks/day)	2	0	0	0
Smoker	Yes	No	Chewed tobacco	No
Drug history	No	No	Yes*	No
Diabetes status	Negative	Negative	Negative	Negative
Cause of Death	CVA Lung Ca	CNS tumour	Accidental trauma	Subdural haematoma
**Hepatocyte characterisation (assay rates)**				
CYP1A2/2A6/2C9/2D6/2E1/3A4 ECOD/UGT (pmol/10^6 cells/min)	>10.0	>10.0	>10.0	>10.0
**Post-thaw hepatocyte viability (*in-house*)**				
Cell viability (trypan blue exclusion)	94%	90%	88%	82%
Number of viable cells (per ml plating media)	56 x 10^4	48 x 10^4	68 x 10^4	36 x 10^4

Cytochrome P450 (CYP) assays performed by Celcis prior/post cryopreservation. Key: BMI, body mass index; CMV, cytomegalovirus; CNS, central nervous system; ECOD, ethoxycoumarin O-deethylase; UGT, uridine diphosphate glucuronosyltransferases; HBV/HCV, hepatitis B/C virus; HIV, human immunodeficiency virus. *marijuana/cocaine.

Supplementary Table 2: Pre-treatment demographic, clinical and biochemical characteristics of study participants.

	Placebo (n=7)	Liraglutide (n=7)	p-value
**Demographics**			
Male sex, n (%)	4 (57.1)	5 (71.4)	1.000
Age (years)	56.0 (39.0-59.0)	59.0 (57.0-60.0)	0.135
Caucasian race, n (%)	7 (100)	7 (100)	1.000
**Metabolic parameters**			
Type 2 Diabetes, n (%)<br>Impaired glucose tolerance, n (%)<br>Normal glucose tolerance, n (%)	3 (42.9)<br>1 (14.3)<br>3 (42.9)	4 (57.1)<br>2 (28.6)<br>1 (14.3)	0.478
HbA1c (%)	5.5 (5.3-6.3)	6.0 (5.6-6.4)	0.244
Pre-study OAD treatment, n (%)	3 (42.9)	5 (71.4)	0.592
BMI (Kg/m^2)	36.5 (29.3-40.0)	34.0 (30.7-35.9)	0.446
Weight (Kg)	101 (85.6-119)	108 (82.5-115)	0.927
Waist circumference (cm)	118 (99.0-127)	116 (102-121)	0.682
Total fat mass (%)	36.2 (28.0-49.2)	33.9 (30.5-36.7)	0.522
Truncal fat mass (%)	41.1 (30.8-49.2)	34.6 (32.3-36.6	0.207
Systolic BP (mmHg)	136 (128-146)	128 (121-133)	0.126
Total cholesterol (mmol/L)	4.50 (4.00-5.06)	4.30 (3.90-5.30)	0.925
HDL (mmol/L)	1.15 (1.00-1.38)	1.12 (0.90-1.30)	0.779
LDL (mmol/L)	3.01 (2.21-3.65)	2.58 (2.40-3.86)	0.966
Triglycerides (mmol/L)	1.68 (1.31-2.12)	1.58 (1.43-1.73)	0.644
TSH (μU/L)	2.80 (1.37-4.06)	2.14 (1.31-2.41)	0.689
Creatinine (μmol/L)	62.0 (57.0-77.0)	71.0 (70.0-89.0)	0.300
Platelets (x10^9/L)	198 (132-222)	211 (178-219)	0.779
**Liver parameters**			
AST (IU/L)	49.0 (34.0-70.0)	64.0 (40.0-87.0)	0.596
ALT (IU/L)	57.0 (20.0-70.0)	90.0 (36.0-137)	0.245
GGT (IU/L)	73.0 (55.0-179)	124 (69.0-183)	0.689
ALP (IU/L)	80.0 (56.0-106)	67.0 (57.0-83.0)	0.603
Bilirubin (μmol/L)	14.0 (6.0-19.0)	12.0 (8.0-14.0)	0.555
Albumin (g/L)	46.0 (44.0-	48.0 (45.0-	0.470

	49.0)	51.0)	
**Liver Histology**			
NAS (/8)	4 (3-5)	5 (4-5)	0.355
Kleiner Fibrosis Stage, n (%)<br>- 0-2 (mild-moderate)<br>- 3-4 (advanced fibrosis/cirrhosis)	 3 (42.9)<br>4 (57.1)	 2 (28.5)<br>5 (71.4)	1.000

Values are median (IQR), unless stated. All blood parameters were in the fasting state. Comparisons of continuous and categorical variables were made with Mann Whitney tests and fisher exact/chi-squared tests, respectively.

Supplementary Table 3: Pre-treatment insulin sensitivity, hepatic de novo lipogenesis (DNL) and serum adipocytokines of study participants.

	Placebo (n=7)	Liraglutide (n=7)	p-value
**Systemic Insulin Sensitivity**			
Fasting glucose (mmol/L)	4.51 (4.43-7.17)	5.48 (4.87-5.61)	0.689
Fasting Insulin (pmol/L)	133 (88.0-220)	98.0 (81.9-109)	0.603
**Muscle Insulin Sensitivity**			
Gd with low-dose insulin (mg/kg/min)	0.68 (0.53-1.27)	0.89 (0.61-0.98)	0.689
Gd with high-dose insulin (mg/kg/min)	3.89 (2.97-4.89)	4.95 (2.49-6.51)	0.872
**Hepatic Insulin Sensitivity**			
Fasting basal EGP (mg/kg/min)	2.03 (1.63-2.41)	2.14 (1.80-2.27)	0.966
Change EGP with low-dose insulin (mg/kg/min)	-1.00 (-1.34- -0.83)	-0.88 (-1.02- -0.78)	0.207
**Hepatic DNL**			
% contribution of DNL to palmitate synthesis (%)	5.24 (4.42-6.90)	4.87 (4.38-5.65)	0.376
**Adipose Insulin Sensitivity**			
Fasting basal NEFA (μmol/L)	421 (397-628)	595 (425-656)	0.966
NEFA with low-dose insulin (μmol/L)	131 (44.6-186)	160 (91.2-204)	0.313
NEFA with high-dose insulin (μmol/L)	15.9 (1.82-36.6)	25.8 (8.51-70.8)	0.689
INS 0.5-MAX NEFA (pmol/L)	180 (106-318)	208 (138-344)	0.872
Adipose-IR index (mmol/L.uU/L)	8.03 (5.34-15.8)	8.42 (5.02-9.88)	0.872
Adipose interstitial fluid glycerol (Basal: AUC μmol/L.h)	382 (215-485)	464 (286-649)	0.446
Adipose interstitial fluid glycerol, low-dose insulin (AUC μmol/L.h)	352 (155-437)	454 (347-504)	0.207
Adipose interstitial fluid glycerol, high-dose insulin (AUC μmol/L.h)	226 (71.3-372)	307 (214-360)	0.522
**Serum Adipocytokines (fasting state)**			
Adiponectin (μg/ml)	6.02 (5.80-8.68)	4.47 (3.68-6.47)	0.038
Leptin (ng/ml)	20.4 (8.25-41.1)	12.7 (10.4-22.8)	0.522
Leptin-to-adiponectin ratio (ng /μg)	3.52 (0.95-6.47)	3.15 (2.11-4.24)	0.779
TNFα (pg/ml)	6.49 (1.19-8.39)	3.90 (3.90-6.49)	0.737
Resistin (ng/ml)	6.34 (6.21-7.58)	5.51 (4.71-6.15)	0.053
Hs-CRP (μg/ml)	5.58 (4.49-7.03)	1.55 (0.63-3.89)	0.073
IL-6 (pg/ml)	4.39 (2.83-5.17)	3.61 (2.83-4.00)	0.226
CCL-2 (pg/ml)	229 (183-275)	210 (203-238)	0.872
CCL-4 (pg/ml)	99.5 (60.2-105)	70.8 (58.6-83.6)	0.513
CCL-5 (pg/ml)	54.4 (40.7-72.4)	65.6 (61.0-77.5)	0.207

Values are median (IQR), unless stated. All variables were collected in the fasting state or during the baseline 2-step hyperinsulinemic euglycemic clamp, isotope tracers and adipose microdialysis. Comparisons of continuous variables were made

with Mann Whitney tests. Key abbreviations: AUC, area under he curve analysis; EGP, endogenous glucose production; Gd, glucose disposal; INS-0.5-MAX NEFA, insulin concentration for ½ maximal suppression of NEFA; TNFα, tumour necrosis factor alpha; hs-CRP, high sensitivity c-reactive protein; IL-6, interleukin 6; CCL, chemokine ligand.

Supplementary figures:

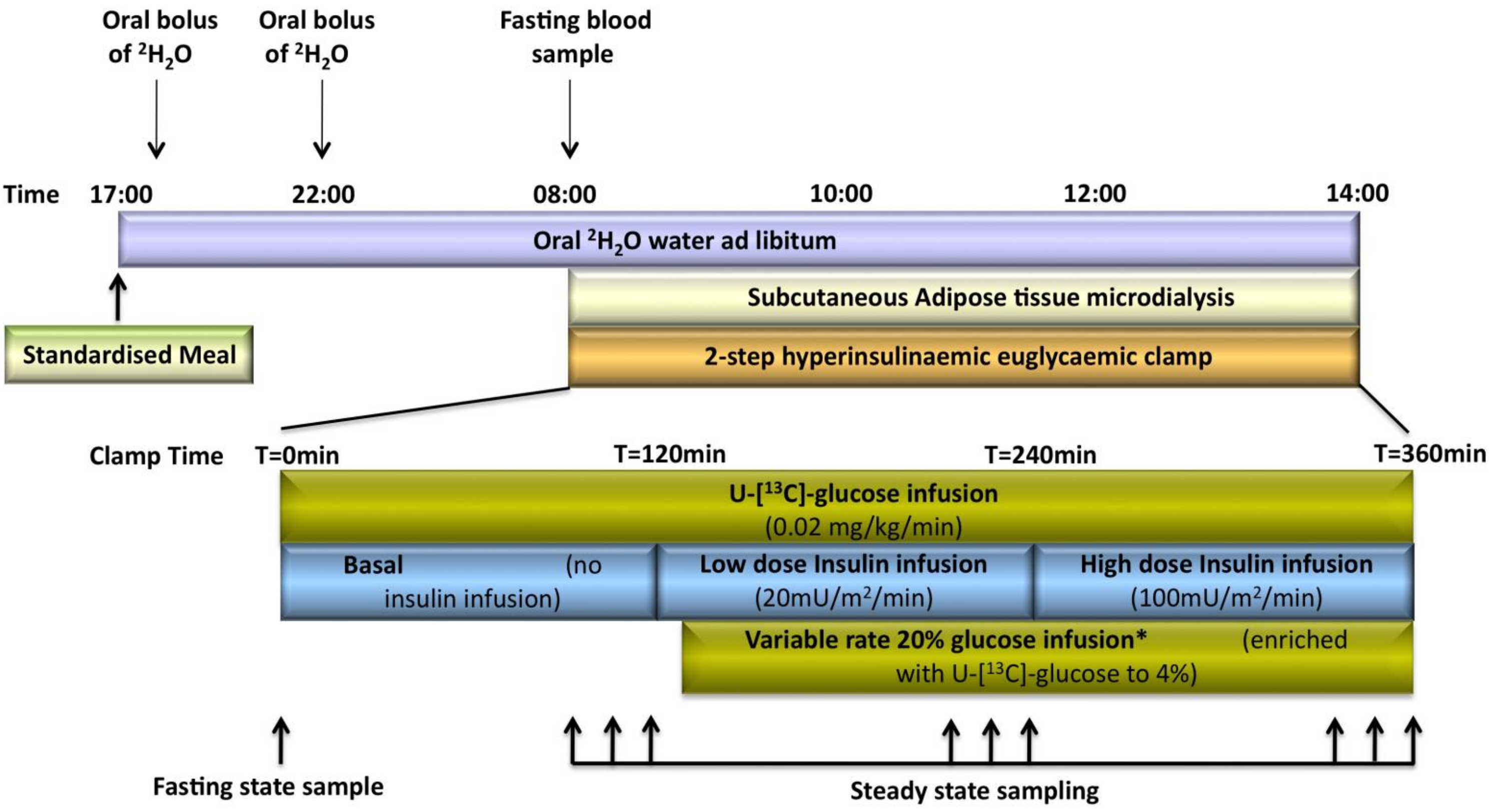

Supplementary figure 1.
Schematic of the 2-step hyperinsulinaemic euglycaemic clamp design. All participants underwent a 2-step hyperinsulinaemic euglycaemic clamp with stable isotope tracers (^{13}C-glucose; $^{2}H_{2}0$ deuterated water) and adipose microdialysis to determine tissue-specific insulin resistance. * variable rate glucose infusion in order to maintain fasting glycaemic control.

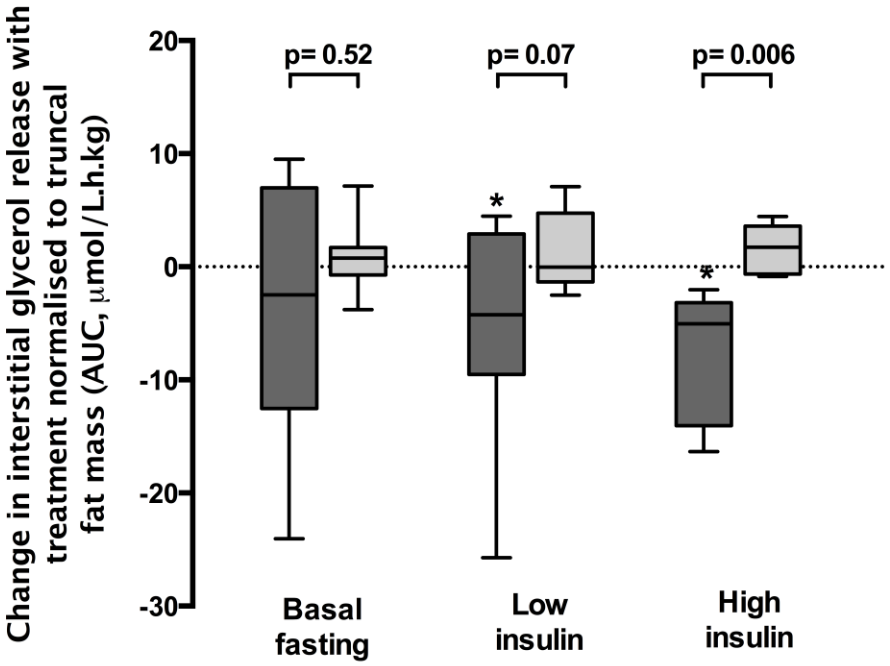

Supplementary Figure 2: Tukey box-and-whisker plots (area under the curve analysis) highlight that liraglutide significantly reduced glycerol release from SAT (after normalisation to changes truncal fat mass on bioimpendance) in response to high-dose insulin compared to placebo, representing decreased abdominal SAT IR. Key: light grey bar = placebo, dark grey bar = liraglutide. *p<0.05 treatment *vs*. baseline (using paired Wilcoxon signed-rank tests). Unpaired mann-whitney tests were used to compare liraglutide *vs*. placebo
